# A qualitative exploration of the experiences of community health animation on malaria control in rural Malawi

**DOI:** 10.1186/s12992-020-00558-3

**Published:** 2020-03-20

**Authors:** Tumaini Malenga, Frances E. Griffiths, Marrit van den Berg, Henk van den Berg, Michèle van Vugt, Kamija Samuel Phiri, Lucinda Manda-Taylor, Eric Umar

**Affiliations:** 1 School of Public Health and Family Medicine, University of Malawi, College of Medicine, Blantyre, Malawi; 2 Training and Research Unit of Excellence, University of Malawi College of Medicine, Blantyre, Malawi; 3 Warwick Medical School, University of Warwick, Coventry, UK; 4 Centre for Health Policy, University of Witwatersrand, Johannesburg, South Africa; 5 Wageningen University and Research, Wageningen, The Netherlands; 6 Academic Medical Centre, University of Amsterdam, Amsterdam, The Netherlands

**Keywords:** Behaviour change communication, Community engagement, Community health animator, Health animation, Health education, Malaria

## Abstract

**Background:**

While great strides have been achieved in fighting malaria through the Roll Back Malaria (RBM) strategy, the recent world malaria report shows an increase in malaria-related deaths compared to previous years. Malaria control tools are efficacious and effective in preventing the disease; however, the human behaviour aspect of the intervention strategies is weak due to heavy reliance on positive human health behaviour. The challenge lies in adoption of control interventions by the target population which, to an extent, may include access to prevention and treatment tools. We present a qualitative assessment of the use of the Health Animator (HA) model for Information, Education and Communication (IEC) to improve adoption and use of malaria control by promoting positive health behaviours.

**Results:**

We conducted 3 Focus Group Discussions (FGDs) and 23 individual in-depth interviews (IDIs) with HAs. Each FGD consisted of 8 participants. Data was analysed using QSR International NVivo 10 software. There are four main themes emerging regarding HA experiences. The perceptions include; collaborative work experience, personal motivation and growth, community participation with health animation and challenges with implementation. Results suggest that HAs were pleased with the training as they gained new information regarding malaria, which affected their use of malaria control interventions within their families. Knowledge was well assimilated from the trainings and influenced personal growth in becoming a community leader. Support from the leadership within the village and the health system was important in legitimising the main messages. The community responded positively to the workshops valued the information imparted. The voluntary nature of the work in a poverty-stricken community affected sustainability.

**Conclusions:**

There is need to empower communities with strategies within their reach. Functioning traditional social support structures are a crucial element in sustainability. Voluntarism is also key for sustainability, especially for rural and remote communities with limited sources of income.

## Background

Considered one of the leading causes of death in the developing world, malaria, a mosquito-borne parasitic infection, affects about 3.2 billion people in 95 countries [1]. Sub-Saharan Africa has the highest burden of the disease, accounting for over 90% of the deaths [1, 2]. Malaria control programs have made substantial progress in recent years to decrease the burden of disease. This has been made possible through the initiation of the Roll Back Malaria (RBM) program, at the start of the new millennium [3, 4]. The RBM program signified global efforts to combat malaria in predominantly low-income malaria endemic countries [3, 5, 6]. The strategy aimed for a coordinated improvement in the management, prevention and treatment of malaria, by increasing access to prevention and treatment tools [3, 4, 7].

While great strides have been achieved in fighting malaria since the inception of the RBM Plan in the year 2000, the recent (2017 and 2018) World malaria reports show a slight increase in malaria-related deaths compared to previous years, which has been attributed to insufficient levels of access and uptake of malaria prevention tools [1, 8].

Malawi has not been spared the devastation, current statistics present it as an area of high prevalence [9]. *Plasmodium falciparum* is the most prevalent parasite responsible for close to 5 million cases annually in a population of roughly 17.5 million people [2, 10]. Much like in other parts of sub-Saharan Africa, there has been a decrease in the number of cases since the adoption and implementation of the RBM strategy for malaria control, the trend however still remains worryingly high [2, 9]. The population most at risk are women and children, and specifically those residing in desolate conditions in remote and rural areas with poor access to preventative and treatment interventions [9–11].

It is well documented that malaria control tools are both efficacious in preventing the disease, however, the human behaviour aspect of the intervention strategies is weak in that the efficiency of such mechanisms rely heavily on positive human health seeking behaviour [12, 13]. The major challenge lies in appropriate adoption of control tools by the target population, which to a large extent includes challenges with accessing prevention and treatment tools [11, 13, 14]. Health information and education campaigns have been dispersed in various forms to encourage and attempt to reinforce positive health behaviours [12, 13]. The RBM plan for disease control now strongly recommends incorporating Behaviour Change Communication (BCC) as an important strategy for malaria control, especially in developing countries with poor health systems [13].

BBC is rooted in socio-cognitive theories on health behaviour. The theoretical model examines the influence of a person’s environment on attitudes, norms and perceptions towards a specific health problem. When used as an intervention, BCC uses advocacy through specific channels of communication to target or influence a person’s intentions towards specific behaviours [15]. BCC works to improve on knowledge of a disease. It stimulates a conversation within the community to promote change in attitude and perceptions of a particular disease. It also works to improve skills on self-efficacy for disease prevention and efficient use of disease prevention tools and health services [10, 12, 13, 16]. The idea behind BCC is to engage a targeted audience into giving them the skills to overcome environmental restrictions and negative perceptions in order to perform the desired act [12, 17]. Although BCC is considerably a novel addition to the RBM strategy, it has been shown to influence health seeking behaviours with the improved communication and importance of using prevention and treatment tools more efficiently [12]. The RBM partnership admits that the BCC tool they prescribe is yet to go through rigorous evaluation, as BCC strategies for malaria control have not been extensively studied [13].

The Health Animation model is a community engagement approach used to promote positive health behaviour for disease control. This is supposed to be achieved by focusing on influencing a mindset of understanding the threat of disease and promoting positive health seeking behaviours [10, 18]. This involves the provision of information through peer-led health education and communication of diseases [10]. The HA approach was pioneered and is promoted by the non-governmental organization, The Hunger Project (THP), for awareness and prevention of disease in developing countries [10, 18]. This concept works within THP’s larger framework of community development through collective accountability and action [18]. The model makes use of a volunteer recruited from within the community, by the community, to train in leadership skills that help unite the community in working towards a common goal, good health and subsequently development [10, 18]. The HA is given leadership training for leading all communal development activities and is provided with training in a condensed version of the science behind illness and disease control. The idea is to then impart knowledge through peer led education, to unite the community in working together for promotion of good health through collective action, most importantly for communicable diseases [10].

The current analysis is based on the Majete Malaria Project (MMP), in Chikwawa District in Southern Malawi [19]. MMP is a multi-stakeholder community-based project for malaria control. Embedded within its community-based activities is an RCT testing malaria intervention in various combinations [19]. The context of this research in presented in the protocol publication by McCann et al. [19]. The implementation of the HA workshops is presented in the publication by Malenga et al. [10] and van den Berg et al. [18]. The MMP project adapted the HA model which THP had previously used for HIV/AIDS awareness and tailored it for malaria awareness and control. The role of the HA in the context of this research was to run community workshops on malaria control in order to influence positive health seeking behaviour of peers in their community [18]. This was to encourage the appropriate adoption and use of malaria control interventions such as Long-Lasting Insecticide-treated Nets (LLIN) and MMP specific interventions of Larval Source Management (LSM) and House Improvement (HI) which were distributed within the community through MMP and the local health system. The workshops were also used as a platform for the community to voice their challenges with malaria control more broadly, and a space to discuss collective solutions to mitigate infection and access treatment. The workshops in this study were run bi-monthly for a period of 2 years prior to data collection. They typically involved the volunteer arranging for a community meeting within their village, where they used a predesigned curriculum to introduce concepts and information on malaria control and the role of all households in committing to compliance with use of interventions for mitigating the burden of disease through collective action [18]. The workshops were interactive, with visual aids and were conducted in the local language, *Chichewa*, for appropriate communication. This process is later followed up with community members reporting a change in health behaviour, which is monitored through the health system in terms of the trend of new infections reported [10, 18].

This paper presents findings of a qualitative assessment of the experience of the HA model as a tool for IEC for malaria control. Specifically, the analysis highlights the perceptions of the trained community volunteers while implementing the HA approach for community engagement and peer influence, to understand the challenges and successes associated with peer led health education for malaria control in rural communities with poor health systems.

## Methods

This study is an exploratory qualitative assessment of the experiences of HA model for malaria control in a rural setting with limited access to health services. Using a total of 3 FGDs and 23 IDIs with HAs, the assessment explored the experiences of community members trained to implement malaria workshops as a mechanism to facilitate learning about malaria and communicating the importance of adopting positive health behaviours to reduce the burden of malaria in their communities. Specifically, the questions asked participants to describe their experience hosting community meetings, working with MMP staff and the support from government health workers. They asked to reflect on their perceptions of malaria behaviour in the community as well as their homes before and after the introduction of the MMP project. They were asked to relay the most obvious changes, and how the MMP project interventions, including the malaria workshops, were received by the community. They were also asked to give feedback on challenges they experienced carrying out the workshops, and what recommendations they would have for future projects of a similar nature.

The context of this research is described in the publication by McCann et al., 2017 [19], and van den Berg et al., 2018 [18]. The implementation of the HA programme is presented in a publication by Malenga et al., 2017 [10], in the Chikhwawa District of southern Malawi. For an in-depth exploration of HA perceptions and experiences, qualitative methods were considered the most appropriate using the BCC model as a guide for data collection and analysis.

### Setting

The research was conducted in communities surrounding the Majete Wildlife Reserve, specifically among those community members who were participating in the randomised control trial of MMP. The Majete Wildlife Reserve is located in the Southern Region of Malawi, straddling 3 districts of Mwanza, Blantyre and predominantly Chikhwawa district. Chikhwawa is a low-lying area in the Shire Valley on the southern end of Malawi, with a population of a little over half a million people. The area is prone to droughts and annual flooding which have substantial impact on food security and livelihoods. Chikhwawa district specifically, accounts for the country’s highest prevalence of malaria. In Chikhwawa, malaria is responsible for 25% of all deaths in the district, where 40–45% of clinical visits and 30 to 40% of all hospital admissions are due to malaria [20].

### Data collection

Participants in both the FGDs and the IDIs were conveniently sampled. They were selected based on their involvement with the project and their capacity as a community-based HA. Participants were identified from a list of HAs registered with the project. The investigator used MMP research assistants to approach the HAs in their homes. The investigator conducted interviews with animators who were available in their home at the time of the visit. A total of 3 FGDs and 23 IDIs were conducted with HAs from the 3 different study sites. Semi-structured interview guides were developed, tested and revised before the data was collected. Each FGD had 8 participants, who were then sampled to conduct the IDIs. All interviews were digitally recorded and conducted in the local dialect, *Chichewa*. The FGDs were conducted in October of 2015 before the commencement of the MMP randomised control trial, and the IDIs were conducted between October of 2016 to February of 2017, 5 months after commencement of the trial.

The study participants included 14 men and 9 women. The median age for men was 30 years, and 37 years for women. The age range for all participants was from 23 to 63 years. All but one of the participants had some primary education, the participant with no education had gone through civic education and therefore possessed basic literacy. Almost two thirds of the HAs had secondary education; none had tertiary education experience. All were married with the exception of two widows. All participants were engaged in subsistence farming, with some complimenting income generation with small local businesses.

### Data analysis

Digitally recorded interviews were transcribed verbatim. They were then translated into English and coded using QSR International NVivo 10 software. Themes emerging from the data were deductively derived from the question guide used in the interviews. Themes were also derived inductively from the data in the transcripts. The analysis explored some features of BCC, by assessing advocacy as a communication strategy to alter individual health belief and the role of peer influence in influencing health behaviour change.

### Ethical considerations

Ethical approval for interviews was given by the University of Malawi’s College of Medicine Research Ethics Committee (certificate number P05/15/1724). All participants agreed to a written consent in the presence of a witness prior to the interview. Participants consented to have interviews digitally recorded.

## Results

There are four main themes emerging from the results regarding HA experiences. The perceptions include; collaborative work experience, personal growth, community response of health animation and challenges with implementation.

### Collaborative work experience

The HAs were generally pleased with their training and the progress of the work they carried out in the community *“With the chief’s involvement it has made the work easy for us to engage with the community…people accepted our message” (IDI).* They indicated that the workload was manageable, through hosting of two meetings a month, covering topics introduced in the training sessions, *‘People have been attending the meetings consistently…they show interest” (IDI).* The HAs also expressed a keen interest in continuing the work independently in future as they were able to appreciate the benefits of such a programme in their community.

*“As health animators we are happy with what we learn. We hope to continue this work even after the project has left. I urge [the project] to continue to teach us more so that we become experts at the end [of the program], and that we are able to run this [malaria workshop] on our own’ (FGD).*

#### Support structures

The HAs considered the village chief an important part of the implementation process. Village Chiefs were involved in the selection of the HA and also supported the running of the workshops. *“To call for a meeting we need to use the chief, without announcing through the chief people would not come to our meeting” (IDI).* The Chief would be responsible for announcing the dates for community workshops and was present at some of the meetings, if unavailable, the chief would send a representative.

The HAs also worked together with government health workers, who took on a supervisory role in the community workshops. Their relationship to government health workers also created a system of accountability, where people visiting the clinic frequently with malaria were referred to attend community workshops in order to learn more about prevention of illness. The Government health workers also assisted in correcting the animator or reinforcing important messages at the community workshops, *“the government [community health] workers help us where we get stuck in disseminating information” (FGD)*. Health talks held at health centres would disseminate information which tallied with lessons learned at the workshops. This gave the community more confidence in the message imparted by the HA in the community. The referrals from the health centres also increased the numbers of participants in the community workshops during the first year of running community workshops.

### Personal motivation and growth

#### Health beliefs

The valuable benefit of the health animation training was the opportunity provided for the volunteers to personally internalise information and understand the real threat of malaria by understanding the main causes, as well as the importance of prevention and minimising the spread of infection within one community.

*“The benefits of this job [community health animation] is that we are now aware of things that we did not know in the past. We now know the importance of rushing to the hospital when sick” (FGD).*

*“Before the project was introduced, when I had malaria, I would take medication up until I felt better. When my health improved, I would stop taking the medication. In a short time, I would get sick again. I would wonder why this happened…but now when I test for malaria and get medication, I complete the dose. I can go months now without getting sick” (FGD).*

The HA felt they had to be convinced on the importance of changing their health behaviour, during their training, before carrying the message forward to the community. *“A community health animator acts as a leader. If you do not lead by example, then it shows as though whatever you are promoting is of no benefit” (FGD).* The HA leading by example, with people following the example, promoted change in health behaviour through the trust nurtured in this new relationship.

*“Before we were made aware, when a person visits the hospital, they would get medication such as LA [Lumefantrine Artemether] and we would share it; if for example I get malaria I would take the medication without finishing it and give the rest to my friend when he gets sick. The project has made us realise that when we receive LA we ought to finish all of it…this shows a change in our lifestyle” (FGD).*

#### Advocacy and peer influence

The general perception from respondents was that the influence of change would first begin with the HA before filtering into the community. The new information learned through the training changed their logic in understanding the disease, and therefore made them more aware of how best to change their behaviour to avoid infection and prioritise treatment.

*“As an animator I am now knowledgeable of a few things I had not known [before]. Some people would deceive me by telling me that if I do hard backbreaking work I will get malaria, or if I get soaked in the rain then malaria follows. Since starting the health animation work, I have seen that it [previous advice] was a lie, malaria spreads with mosquitoes. I have also learnt that it’s not all mosquitoes that spread malaria” (FGD).*

Through leading by example; adopting desired disease prevention and treatment health behaviour they were also able to appreciate the socio-economic benefits of minimising illness in the household.

*“The benefits of animation work… from the training I received I am able to recognise what malaria is; how it starts and how it spreads and how I can personally avoid [infection]. It [health animation training] has helped me personally in my household with improving my life because of having less malaria, because I follow what I was taught…instead of wasting time at the hospital dealing with this disease” (FGD).*

Their ability to influence their peers consequently assisted with dispelling false rumours of the origins of the disease, that perpetuate illness by promoting poor health seeking behaviour. The HA role helped the volunteers to lead by example in terms of managing malaria within their household.

### Community participation

With the introduction of the community workshops, people were very eager to attend the meetings and appeared in large numbers proportional to the village population. From attending the workshops, community members were able to share what they learned with friends in their community, carrying the message along to those who were not attending the meetings. At times this also served as a way of encouraging others to participate in the workshops.

*“In the beginning we would hold our meetings in the community, we would include plays to encourage people to attend the meetings. Those that missed the first meeting would show up to the next. They would ask questions…this was because malaria was causing problems [for them] and they wanted to understand [the problem]” (FGD).*

*“When a person attends a meeting and learns the benefits of taking medication or going to the hospital, they would take the message to their friends in the community to tell them what they were taught at the workshop…their friends would ask when the next meeting was so that they hear the message for themselves” (FGD).*

The HAs required some level of creativity to maintain interest in their audience, as well as to encourage attendance. They would use theatrical performances as a way of making the lessons interactive. During the training they were encouraged to recruit audience members to perform a candid skit after each lecture. This was meant to display context of the lesson to the audience and was also used as a way of assessing how well the audience had understood the key message.

*“We would use drama, and choir to get people to attend the meetings and to participate. We would select people from within the community and give them roles in a play with guidance and a topic” (FGD).*

The scenarios used relayed the message of making positive choices on health seeking behaviour. This was designed to allow people to understand the importance of making the right choice as well as the implications of wrong decisions with regards to disease prevention and treatment.

In some instances, there was distrust in the community on the intentions of the research activities within the community. There was very little understanding on the objectives and intentions of the MMP randomised control trial. The HA acted as their point of contact with the MMP as well as the source of information where necessary.

*“We had community members who did not take part in the workshops. When the research started [enumeration], people were then able to see the connection between our work and the research project. That is when we noticed more people attending the meetings, once they realised, we are connected to the project” (FGD).*

*“The community had a problem with the people putting the stickers on the door…they did not trust them; they would say they had other intentions that they were not disclosing. But as health animators we were able to explain properly the intentions of the project. People were then willing to accept the project and were then looking forward to receiving bed nets” (FGD).*

### Implementation challenges

#### Community Engagement

In general, people showed interest in the community meetings. However, there were some factors that affected attendance. *“As you know, at the moment there is a problem of famine, and although we get handouts, people rush for piece work in the gardens” (IDI*). During times of hunger or planting season, people would prioritise their farms and getting food over attending a meeting. Funerals were also prioritised over community meetings.

*“Other times very few people came, especially if the meetings coincided with other activities... For example, if we held a meeting on the day there was a football match, that meant that we shared the crowd and we would have fewer people attending,” (IDI).*

At times the HAs struggled with relating their teachings to services in the health system. In as much as the community was willing to change behaviour, the barrier existed when the health system was failing to provide the services that would encourage the community to reinforce the positive health behaviour change.

*“We taught them about not using medication without getting a malaria test. But the problem is that some clinics that we have in the community, the doctors give medication without giving a test to check whether the person has malaria or not. This is making people ask ‘I thought you told us that we should only get medication when we have been tested for malaria? Why is it that these clinics do not have the equipment to test for malaria? we are receiving malaria medication without being tested’, yes” (FGD).*

The MMP community is set in a disaster-prone area with crippling poverty. This created some problems in the community’s understanding of research and the intentions of the workshop for malaria. People expected to be given donations or benefit with more tangible items other than knowledge. This attitude was a result of receiving handouts from charity and governmental organizations in times of disaster.

*“Some of the people say, ‘the project is useless. Whatever you promised to happen has not been done, so now we cannot listen to anything you have to say’. The chief would announce our meetings and people would not come. We would get maybe three people, but we still teach them because we need to do our job” (FGD).*

The community was set to receive bed nets as part of the universal coverage of LLINs for the control arm of the RCT intervention. Due to logistical delays, the nets had not been delivered for over 18 months from the proposed date. The delay affected the commitment of the community to consistently attend meetings.

*“Despite the challenges, we still feel this project has benefited the community. Even though nets have not been distributed, there are people in the community that own nets but don’t use them. Some would hold on to their nets and not use them expecting to use nets from the project. We would explain to them that they should still use the nets because between now and when nets are distributed, they could get sick, and it will be a problem for them not the project. Those who understood would [start to] use them” (FGD).*

#### Attendance

Attendance was substantial during inception and increased gradually the first year of implementation. However, during the second year of the program, the numbers declined, making it difficult for some HAs to maintain the meetings consistently. The community did start to experience participation fatigue with the community workshops.

*“In the beginning things worked very well and people had accepted [the programme] well based on attendance, I felt the work was progressing well…Currently, as animators we feel the work is good, but the community are tired of hearing the same message being delivered to the same group of people” (IDI).*

The problem of declining attendance was largely due to the repetitive nature of the lessons or lack of new information in the second year, community members felt that they had assimilated the information well the first time and saw no need to be reminded or take the time to attend the workshops. The decreasing attendance forced the HAs to deviate from their prescribed engagement and communication methods. They would resort to some level of coaxing for participation, by promoting the idea of bed net distribution as an incentive to participate in the research. This would later lead to a coerced attendance of workshops.

*“we would encourage them to attend the meetings. When they realised the link we had with the project we would explain to say that if they wanted to participate in the project, to receive the net, then that process would start here [attending workshops]. If they do not attend the workshop, then they would not participate in the research and would then not receive the net” (FGD).*

*“When we have problems with some communities, we sit down to discuss how we can approach that community…we are being creative and coming up with new ideas on how to approach people, at times raise money to give them a little entertainment so that we entice them to the meetings…there are challenges but with this program people can change [their behaviour]” (FGD).*

With delays in implementing the net distribution, this coercion resulted in a drop in attendance of workshops as whatever was perceived as promised was not delivered to the community. As a result, the community felt there was no benefit in attending the workshops or engaging with the health messages delivered.

*“At the start of our programme, we as health animators, would receive respect [from the community]. We would talk and people would listen. But now people are not willing to listen. They say that in the beginning they felt the project started off strong, but now it is weak, and they wonder why. We are not receiving respect…we need new ideas, to bring something different to the people so that they feel it’s a new thing so that they come back [to attend meetings], because right now we have lost them” (FGD).*

*“The strategy we are using now is that when the chief calls for a meeting, we take advantage of it. We tell them that we would like to give talks…the chief would say that when he is done with his meeting, we can then address the people. This is working, and this is the system we are now using. But if we tell the chief to call an independent meeting for our workshop in the community the people would say ‘that meeting is useless, a project with no benefits’ so we are being insulted for the work” (FGD).*

#### Volunteerism

In terms of commitment to working as an animator, the concept of volunteerism was difficult to accept and manage in this rural and indigent context. The role of the animator was still understood as a type of formal employment, one that specifically doesn’t pay, *“They say when you plant a tree, for it to live it must be watered. I think it would be good if this project had a way of engaging us frequently to keep us confident” (FDG 003 CHA).* The cost of foregoing labour, especially for subsistence farmers, to run animation workshops, came at a significant cost to the household. This was most evident during the cultivating season as it limited what time they were able to dedicate to their role of generating income within their household.

*“The activities of [animation] work and household work can be challenging when trying to manage time…it can be a problem because we do not get anything for it. It seems to be slowing us down a bit. Because we do not get trained frequently, we may forget we are animators” (FGD).*

HAs were also mocked for taking on ‘employment’ without remuneration, it was seen to some of their friends as a waste of time that could be spent making money for the family. *“The challenge with this job is that we work with no pay. We work without receiving anything because it’s voluntary” (FGD).* At times this came with the consequence of the loss of respect from their peers in the community which also impacted the efficiency of their work for imparting knowledge to the community.

*“We are told that we are leaving productive jobs to do something with no [financial] benefit ‘the time you are spending here, we are spending in the field farming’…Because we opted to do the work, we still continue to do it” (FGD).*

Most of the animators expressed their concern on the loss of income and suggested some form of payment, however minimal, to cover the cost of foregoing labour for their households.

*“As a woman it presents some challenges, I have to tell my husband that I am going to work so he will be alone working in the field. At times he will tell me the days are too many and I am not contributing much to the home. I do apologise and continue doing the [animation] work” (FGD).*

The limitation on income at times created the situation where animators were forced to migrate to seek employment, which meant they were unable to run workshops in the community, *“at times we feel it’s better to go across to Mozambique to do some piecemeal work instead of running workshops…we feel neglected” (FGD).* At the start of the program they did admit to accepting the role and understood the nature of voluntary work. However, they assumed that they would be trained frequently. The frequent training would mean access to a monetary allowance for attending the training. It was assumed this would be the alternative source of income if they would be foregoing labour dedicated to other modes of generating income.

*“Our friends are in small projects where they get a budget [money] and they say ‘oh well you are an animator’ and we would not be given other roles [in community work]. It’s a big problem…We are not paid; we are struggling to pay fees…the man of the house alone cannot do everything. Maybe in future they [the project] can consider that” (FGD).*

When the opportunity for monetary allowances was not available to them, they were forced to seek employment elsewhere or find alternatives for income generation. This meant the running of workshops was inconsistent and at times incomplete. This situation forced them to falsify reports; a requirement for them to maintain their role as a HA.

*“We go to Mozambique for work and not run the workshops. When they ask us for reports [from workshops] we can write false reports. This is because we are not monitored or supported adequately” (FGD).*

## Discussion

This study explored the experiences of being a HA in a rural context. The HA model for community engagement can be used effectively as a form of IEC to complement malaria control interventions. This strategy may very well improve efficiency of implementation and use of malaria control interventions in rural communities. The simplified science behind malaria control delivered through HA workshops increases the knowledge on exposure and risk of infection. This could therefore influence people to use malaria interventions more appropriately and consistently as their understanding of the threat of disease and the role of the intervention is made clearer. It is a useful intervention as a form of advocacy, by providing key messages that are accessible to the community and providing information on how to overcome contextual barriers in preventing and seeking treatment for malaria.

The results illustrate community HAs’ personal experiences in carrying out malaria workshops. They present the various strengths and weaknesses of health animation when working in the context of rural and remote communities with poor access to health services. The study also presents the contextual challenges encountered with implementing animator workshops in a community with a history of high dependency on aid and how that can strongly jeopardise the objectives of introducing this form of information dissemination for health behaviour change.

The valuable benefit of the health animation training was the opportunity provided for the volunteers to personally internalise information and understand the real threat of malaria by understanding the main causes, as well as the importance of prevention and minimising the spread of infection within one community. If leading by example, there is some potential for a community to benefit from peer influence for health behaviour change. The HAs were able to make use of malaria prevention interventions and noted the personal gains within the household. Less illness translated to less time and finances spent seeking care and more time spent on improving their livelihood through the gains in time dedicated to labour for socioeconomic benefits. There was a notable cost of volunteering through missed work opportunities, however, this was acceptable to those choosing to continue the work.

The study demonstrates that it is feasible to utilize the HA model for community engagement with malaria control if the volunteer feels well supported by the implementing partners [10, 18]. The context of this research shows it is feasible to introduce Health Animation in a rural set up [10]. However, characteristics of the population strongly influence how successfully the programme may be implemented [21]. This suggests the need for adaptation to deal with challenges presented by context [22]. Interactive sessions for introducing medical and health concepts, and facilitating the interpretation and application of these concepts to generate knowledge, as used in this study, have been shown to be more fruitful for influencing behaviour change [23]. The challenge lies in sustainability of any change in behaviour in the long term [10, 16, 23]. Knowledge and attitude change are generally possible through provision of concepts, information and tools [21, 24–26], however the maintenance of the change in desired behaviour may require a lot more input than simply communicating health information [21, 23].

Contextual variables can pose major challenge in implementing projects for HAs. The widespread poverty in the catchment area, coupled with the fact that it is a disaster-prone area created problems of implementation in terms of understanding the intentions of the project and the workshops for malaria control. The community expected handouts in exchange for participating in malaria workshops and conversing with HAs. Once it was clear there was no material benefit attendance of the workshops declined significantly. The problem of poverty also created a challenge in successfully internalising the intentions of the HA program as well as the importance the malaria workshops played in mitigating disease within the community. For the purposes of health information dissemination, it would therefore have to be delivered differently in this context. Additionally, if the HA programme were to be introduced as part of the health system, a form of remuneration would ensure some level of sustainability, as commitment to carrying out the workshop would increase, both in terms of the HA taking pride in having a title and formal employment, as well as a source of income for their family.

The initial pull to attend malaria workshops stemmed from the community’s curiosity to discover what the HA had learned from their trainings. They were also curious to find out what the project was about after discovering the link to the research under MMP. The HA was useful in debunking myths and standing in as a source of information for the community, when information would have been otherwise unavailable. This makes them key individuals, especially in the context of implementing health research projects with some degree of complexity. Repetition of modules when delivering the workshops meant that with time attendance declined. There is therefore the need to diversify the curriculum keeping it current or revising it with new information as and when changes are made with the health system policy. Alternatively, the delivery could be revised to make it more engaging considering the context it is delivered in.

Community leadership, through both the village chiefs and health system workers, has a strong influence in the success of such interventions. HAs felt the influence of the chief was important for enticing the community to attend workshops. The involvement of village chiefs and health systems representatives legitimises the intervention and promotes engagement with the community [23]. The chiefs in this programme endorsed and promoted the malaria workshops as part of the larger efforts of municipal development in their communities.

## Limitations

The limitations of this study are; firstly, the data collection was carried out by a member of the project, which may have influenced the quality of data generated as impartiality was affected. Secondly, the timing of the data collection was done early into the randomised control trial and may present part of and not the whole spectrum of experiences for the animator. Lastly, the data was limited to qualitative interviews and would benefit more from a broader survey where results can be generalised.

## Conclusion

Community Health Animation is a viable option for disseminating information on malaria control for communities with limited access to health services. However, the livelihood challenges existing in the local context; such as poverty and unemployment, poor health service infrastructure and access to health services, influence the implementation and success of the HA programme for health behaviour change. There is need to create a curriculum that is interactive, context specific and diverse to consistently engage the audience for consistent attendance. Functioning traditional social support structures are crucial element of sustainability. It is also important to note the effects of good leadership and local governance structures within the community to ensure sustainability, more important in rural indigent communities. Further research should focus on how BCC influences health behaviour change and its sustainability in the longer term.

## Data Availability

The datasets used and/or analysed during the current study are available from the corresponding author on reasonable request.

## Abbreviations

BCC: Behaviour Change Communication
FGD: Focus Group Discussions
HA: Health Animator/ Animation
IDI: In-Depth Interviews
IEC: Information Education Communication
LLIN: Long Lasting Insecticide-treated Nets
LSM: Larval Source Management
MMP: Majete Malaria Project
RBM: Roll Back Malaria Plan
THP: The Hunger Project
